# Association of alcohol and drug use with use of electronic cigarettes and heat-not-burn tobacco products among Korean adolescents

**DOI:** 10.1371/journal.pone.0220241

**Published:** 2019-07-31

**Authors:** Yeji Lee, Kang-Sook Lee

**Affiliations:** 1 Department of Public Health, Graduate School, The Catholic University of Korea, Seocho-gu, Seoul, Korea; 2 Department of Preventive Medicine, College of Medicine, The Catholic University of Korea, Seocho-gu, Seoul, Korea; Leibniz Institute for Prevention Research and Epidemiology BIPS, GERMANY

## Abstract

**Background:**

The tobacco industry has aggressively introduced new and diverse products in the market, including electronic cigarettes (e-cigarettes) and heat-not-burn (HNB) tobacco products, to which adolescents are readily susceptible. Conventional cigarettes have a well-established relationship with adolescent risky behaviors such as alcohol and drug use; however, no studies exist on the association between alcohol consumption and use of e-cigarettes or HNB tobacco products among Korean adolescents. This study evaluated alcohol-related behaviors and drug use in relation to whether a Korean nationally representative adolescent sample had ever used e-cigarettes and HNB tobacco products.

**Methods:**

Data from the 2018 Korean Youth Risk Behavior Web-based Survey were analyzed. The final study sample comprised 60,040 adolescents. Chi-square and logistic regression were used to examine whether the ever having used e-cigarettes and HNB tobacco products was associated with alcohol-related behaviors and drug use. P-values for trends were calculated to examine the dose-response relationship for each variable.

**Results:**

Respondents with higher drinking frequency, drinking quantity, alcohol intoxication, alcohol availability, and drug use were more likely to report having used e-cigarettes and HNB tobacco products, thus implying a significant relationship between substance use and novel tobacco product (P < .001).

**Conclusions:**

Our findings suggest that at-risk adolescents who are engaged in other forms of risk-taking behaviors are prone to attract the experimentation with e-cigarettes or HNB tobacco products. Thus, smoking cessation programs related to substance use should be implemented, and there is an urgent need to monitor and regulate these products effectively.

## Introduction

The marketplace of tobacco products has been in a dynamic state and has changed significantly in recent decades with the emergence of new tobacco products, including electronic cigarettes (e-cigarettes) and heat-not-burn (HNB) tobacco products. E-cigarettes are battery-powered devices that deliver nicotine to the user through vaporization of a propylene-glycol solution, typically in combination with flavors [1]. HNB tobacco products are cigarettes that use electronic devices to generate nicotine vapor by heating solids to high temperatures without causing combustion [2]. As the recent tobacco industry aggressively introduces new and diverse products in the market [3], adolescents are susceptible to use these new products, as they readily become interested in them [4]. The prevalence of e-cigarette use among adolescents has increased in many countries including the US [5], UK [6], France [7], Canada [8], and Poland [9]; recent US estimates indicate that use of e-cigarettes in the past 30 days has surpassed the use of regular cigarettes [5]. In Korea, ever-use and current use of e-cigarettes among adolescents increased from 7.4% to 7.9% and 2.2% to 2.7%, respectively, between 2017 and 2018 [10]. Moreover, the awareness and use of HNB tobacco products have increased dramatically since they were first launched [11–14]. For example, a study of Japanese adolescents found that almost half were aware of HNB products and one fifth had already tried them [11]. An Italian study showed that almost 30% of respondents were aware of HNB products and 1.4% had tried them [12]. Awareness and use of HNB tobacco products have increased among US adults, especially men and younger adults [13]. Results of recent research indicated that awareness, experience, and current use of HNB tobacco products have rapidly increased among Korean young adults, with respondents believing that HNB tobacco products would help them to quit smoking and are less harmful to their health than conventional cigarettes [14]. Tobacco companies have competed in launching a variety of HNB products that are appealing to adolescents for their high-tech appearance, while leading users to believe that HNB products are less harmful than conventional cigarettes [15, 16]. The number of heated cigarettes sold accounted for 9 to 10% of the total Korean tobacco market share in 2018 [17]. A recent study suggested that there was great public interest in HNB tobacco products, and the use of this type of product will increase [18]. The rapid market growth and soaring popularity of HNB tobacco products are a growing public health issue across the world, yet little is known about the epidemiology of their use among adolescents.

It was reported that half of the Korean adolescents (42.3%) had the experience of alcohol consumption in 2018 [19]. Alcohol drinking can give a negative health impact such as cancer, hypertension, coronary heart disease, injuries, and the difficulty of academic achievement [20, 21]. Moreover, the prevalence of binge drinking among adolescents who drink alcohol has increased from 44.6% in 2008 to 52.5% in 2018 [19]. Binge drinking among adolescents can lead to addiction, alcohol dependence, drug use, unplanned sexual experience, and other serious health problems [22–24]. It was also reported that heavy alcohol consumption can occur more risk of violence [25]. In addition, alcohol intoxication is associated with increasing an aggressive response [26], and alcohol availability was also related to harmful behaviors such as violence occurring and alcohol related-harms [27].

Conventional cigarettes have a well-established relationship with adolescent risky behaviors such as alcohol and drug use [28–30]. With the growing recognition of e-cigarettes, studies in several countries support a strong association between e-cigarette use and alcohol/drug use among adolescents. A recent US study found that adolescents who drink alcohol frequently are more likely to have used e-cigarettes [31]. Having ever engaged in binge drinking or use of marijuana was found to be predictors of ever having used e-cigarettes among adolescents in New Zealand [32]. However, to date, studies on the association between alcohol and e-cigarette use have not been conducted in Korea, and the use of HNB tobacco products among adolescents has not been examined. Considering the similarity between tobacco and HNB tobacco products, the relationship between the use of HNB tobacco products and substance use needs to be addressed.

The aim of this study was to examine the association between ever having used e-cigarettes or HNB tobacco products and alcohol-related behaviors and drug use. As previous studies of this issue were predominately conducted in Western countries, we used a Korean nationally representative adolescent sample to evaluate how alcohol-related behaviors and drug use affect ever having used of e-cigarettes. Moreover, we also conducted analysis to examine for the first time how alcohol-related behaviors and drug use affect use of HBN tobacco products.

## Methods

### Study population

The present study was based on secondary data drawn from the Korea Youth Risk Behaviors Web-based Survey (KYRBWS) [19]. Since 2005, the Korea Centers for Disease Control and Prevention (KCDC) have conducted this online survey every year using a self-administered questionnaire to identify health-related behaviors, including smoking, alcohol use, obesity, dietary behaviors, and physical activity among secondary school students in Korea [33]. The purpose of the KYRBWS is to generate statistical data on health risk behaviors of Korean adolescents and to use the data to plan and assess health promotion programs designed for Korean adolescents [33]. In the 2018 KYRBWS, the questionnaire contained 103 items in 15 domains of health risk behaviors (e.g., smoking, alcohol use, and physical activities), and 97 indices were calculated [19]. The questionnaire items and indices were developed based on national and international literature as well as expert consultations [19, 33]. To verify the applicability of the surveys, the understanding, reliability and validity of each question were investigated by the KCDC [34]. Each question was considered reliable and valid [34, 35]. Especially, the estimates of smoking related variables revealed a good validity [34]. The survey was conducted over the Internet using an anonymous, self-administered questionnaire, which was completed on computers in a computer lab [33]. The target population of the 14th KYRBWS (2018) comprised students attending secondary schools across Korea as of April 2018 [19]. A complex sampling method was used to select a sample representing the target population [19, 36]. The sample selection process involved stratification, sample allocation, and sample extraction. At the stage of stratification, to minimize sampling errors, the population was divided into geographic regions and types of schools. For sample allocation, a total of 400 middle schools and 400 high schools were set as the sample size to ensure adequate population composition. Sample extraction was then executed using stratified cluster sampling, with the first extraction unit being school and the second extraction unit, class. A total of 60,040 students completed the survey, resulting in a participation rate of 95.6% [19]. This high level of response rate was possible, because the survey was administratively conducted with administration cooperation of the Korean Ministry of Education [33]. In addition, the KYRBWS did not permit missing responses, because the survey’s software would only show the next question if the current question had been answered [19]. Variables with logical errors and anomalies were treated as missing values in the supplied raw data, but the variables used in the current work had no missing values; thus, all 60,040 students were included in the analysis.

This study utilized government-approved statistics by KCDC, and all students who participated gave informed consent. This study was approved by the institutional review board of the Catholic University of Korea (approval no. MC19ZESI0005) to analyze secondary data of the 2018 KYRBWS.

### Study variables

#### General characteristics

We used the following socio-demographic factors and cigarette smoking status as control variables in the model: gender (male/female), school grade (middle 1st/middle 2nd/middle 3rd/high 1st/high 2nd/high 3rd), self-perceived academic achievement (high/middle-high/middle/middle-low/low), and perceived economic status (high/middle-high/middle/middle-low/low). Cigarette smoking status was categorized into three groups: current (participants who had smoked conventional cigarettes in the last month), ever (participants who had ever smoked conventional cigarettes but not in the last month), and never (participants who had never smoked conventional cigarettes in their lifetime).

#### Ever-use of e-cigarettes and HNB tobacco products

Whether respondents had ever used e-cigarettes was identified through a “Yes” response to the yes/no question “Have you ever used e-cigarettes?” Ever having used HNB tobacco products was identified through a “Yes” response to the yes/no question “Have you ever used HNB tobacco products (IQOS, glo, or Lil)? Conventional cigarettes and e-cigarettes are excluded.”

#### Alcohol-related behaviors

Drinking frequency was measured using the question, “How many days have you drunk more than one alcoholic drink in the last 30 days?” The responses were categorized into four groups: non-drinker (no consumption of alcoholic drinks during the last 30 days), 1–5 days (consumption of alcoholic drinks between once and five times per month), 6–9 days (consumption of alcoholic drinks between six and nine times per month), and ≥10 days (consumption of alcoholic drinks more than 10 times per month). Drinking quantity was assessed using question, “When you drank, what was the average amount of alcohol you drank per drinking session in the last 30 days?” The responses were categorized into four groups: non-drinker (no consumption of alcoholic drinks during the last 30 days), ≤2 bottles of beer (consumption of up to two bottles of beer, or less than four glasses of Soju, a Korean distilled spirit), 3–4 bottles of beer (consumption of more than two but not more than four bottles of beer, or five glasses to two bottles of Soju), and >4 bottles of beer (consumption of more than four bottles of beer, or at least two bottles of Soju). Alcohol intoxication was measured using question, “How many days have you drunk enough to lose your mind or consciousness during the last 30 days?” The responses were categorized into four groups: no-intoxication (non-drinker or drinker without experience of intoxication), 1–2 days (intoxication between once and twice per month), 3–4 days (intoxication between three and four times per month), and ≥5 days (intoxication more than five times per month). Alcohol availability was assessed using the question, “In the past 30 days, when you tried to buy a drink at a convenience store or a store, how easy was it?” The responses were categorized into four groups: no-attempt (no attempt to buy alcoholic drinks during the last 30 days), impossible (impossible to buy alcoholic drinks), with effort (possible to buy alcoholic drinks with effort), and without effort (possible to buy alcoholic drinks without any effort).

#### Drug use

Drug use was assessed with the question, “Have you ever used drugs, inhaled butane gas, or sniffed glue habitually or intentionally in your lifetime?” Based on their answers, participants were categorized into three groups: current (“I often use drugs these days.”), ever (“I have used drugs before, but I do not use them these days.”), or never (“None.”).

### Statistical analysis

Statistical analysis was performed using SPSS 25.0 (IBM, Armonk, NY, 2017). Complex sampling weights were applied to reflect nationally representative samples. A chi-squared test was performed to examine having used of e-cigarettes and HNB products according to the general characteristics of participants. A chi-squared test was also performed to assess alcohol-related behaviors and drug use of participants by cigarette smoking status, and having ever used e-cigarettes and HNB products. A multiple logistic regression analysis was performed to examine the association of ever having used e-cigarettes with alcohol-related behaviors and drug use. A multiple logistic regression analysis was also performed to examine the association of HNB product use with alcohol-related behaviors and drug use. Each variable of pertaining to alcohol-related behaviors and drug use that was used as an independent variable entered the logistic regression model separately, after adjusting for gender, grade, perceived school performance, perceived economic status, and smoking status. Ever having -used of e-cigarettes and HNB products were used as the main outcome variables. According to the Variance Inflation Factor (VIF) values, every variable had values lower than 10, so there were no collinearity problems. P-values for trend were calculated to examine any dose-relationship with the drinking frequency, drinking quantity, the frequency of alcohol intoxication, the availability of alcohol products, and drug use. Nagelkerke R^2^ was used to estimate the amount of variance explained. Adjusted odds ratios (AORs) and the corresponding 95% confidence intervals were calculated. The significance level was set at P < .05.

## Results

### Participants’ general characteristics

General characteristics of the participants are shown in Table 1. A total of 12.3% of male and 3.1% of female adolescents had ever used e-cigarettes, and 4.4% of male and 1.2% of female adolescents had ever used HNB tobacco products. The proportions of both ever having used e-cigarettes and ever having used HNB tobacco products increased as school grade increased. Among those who had low self-perceived academic achievement, the proportion who had ever used e-cigarettes (18.0%) was more than twice that among those with high self-perceived academic achievement (6.6%). Among those with low self-perceived academic achievement, the proportion who had ever used HNB tobacco products (7.1%) was also more than twice that among those with a high self-perceived academic achievement (2.9%). The proportion who had ever used e-cigarettes among those with low perceived economic status (17.9%) was almost twice that among those with high perceived economic status (9.7%). Similarly, ever having used HNB tobacco products among those with low perceived economic status (8.4%) was almost twice that among those with high perceived economic status (4.2%). Moreover, nearly two thirds (65.2%) of current smokers reported ever having used e-cigarettes and nearly one third (32.4%) reported ever having used HNB tobacco products.

**Table 1:** Participants’ general characteristics (N = 60,040)^1^.

Variables	Category	Never used e-cigarettes	Ever used e-cigarettes	P	Never used HNB tobacco products	Ever used HNB tobacco products	P
		n (%)	n (%)		n (%)	n (%)	
Gender	Male	26,985 (87.7)	3478 (12.3)	< .001	29,229 (95.6)	1234 (4.4)	< .001
	Female	28,689 (96.9)	888 (3.1)		29,243 (98.8)	334 (1.2)	
School grade	Middle school 1st	9738 (98.9)	109 (1.1)	< .001	9823 (99.7)	24 (0.3)	< .001
	Middle school 2nd	9730 (96.1)	362 (3.9)		9987 (98.9)	105 (1.1)	
	Middle school 3rd	9693 (94.0)	597 (6.0)		10,137 (98.3)	153 (1.7)	
	High school 1st	8412 (90.6)	848 (9.4)		8943 (96.6)	317 (3.4)	
	High school 2nd	8936 (88.9)	1103 (11.1)		9650 (96.2)	389 (3.8)	
	High school 3rd	9165 (86.5)	1347 (13.5)		9932 (94.1)	580 (5.9)	
Self-perceived academic achievement	High	7584 (93.4)	485 (6.6)	< .001	7858 (97.1)	211 (2.9)	< .001
	Middle-high	14,655 (95.1)	696 (4.9)		15,119 (98.4)	232 (1.6)	
	Middle	16,518 (93.7)	1008 (6.3)		17,205 (97.9)	321 (2.1)	
	Middle-low	12,052 (90.4)	1197 (9.6)		12,830 (96.6)	419 (3.4)	
	Low	4865 (82.0)	980 (18.0)		5460 (92.9)	385 (7.1)	
Perceived economic status	High	5958 (90.3)	568 (9.7)	< .001	6274 (95.8)	252 (4.2)	< .001
	Middle-high	16,552 (93.2)	1129 (6.8)		17,276 (97.5)	405 (2.5)	
	Middle	25,998 (92.9)	1810 (7.1)		27,214 (97.6)	594 (2.4)	
	Middle-low	5966 (89.9)	616 (10.1)		6372 (96.6)	210 (3.4)	
	Low	1200 (82.1)	243 (17.9)		1336 (91.6)	107 (8.4)	
Cigarette smoking status	Current	1375 (34.8)	2347 (65.2)	< .001	2544 (67.6)	1178 (32.4)	< .001
	Ever	3462 (71.0)	1356 (29.0)		4579 (94.8)	239 (5.2)	
	Never	50,837 (98.6)	663 (1.4)		51,349 (99.7)	151 (0.3)	

^1^Weighted percentages following complex sample analysis.

HNB, heat-not-burn.

### Alcohol-related behaviors and drug use according to cigarette smoking, and ever having used e-cigarettes and HNB tobacco products

Differences in alcohol-related behaviors and drug use according to cigarette smoking status, and ever having used e-cigarettes and HNB tobacco products are presented in Table 2. Rates of current cigarette smoking, and ever having used e-cigarettes and HNB tobacco products were highest in participants who drank alcohol more than 10 days per month, followed by those who drank 6–9 days per month and 1–5 days per month (P < .001). In addition, rates of current cigarette smoking, and ever having used e-cigarettes and HNB tobacco products were highest in participants who drank over four bottles of beer, followed by those who drank more than two but not more than four bottles of beer, and those who drank two or less bottles of beer. Rates of current cigarette smoking, as well as ever having used e-cigarettes and HNB tobacco products, were highest in participants who experienced alcohol intoxication more than 5 days per month, followed by those who experienced alcohol intoxication 3–4 days per month and 1–2 days per month (P < .001). In addition, rates of current cigarette smoking and ever having used e-cigarettes and HNB tobacco products were highest in participants who were able to buy alcohol without any effort, followed by those who were able to buy alcohol with effort, and those who were unable to buy alcohol. Rates of current cigarette smoking, and ever having used e-cigarettes and HNB tobacco products were also highest in participants who were current drug users, followed by those who had ever used drugs.

**Table 2:** Alcohol-related behaviors and drug use according to cigarette smoking status and ever-use of e-cigarettes and HNB tobacco products (N = 60,040)^1^.

Variables	Never smoked cigarettes n (%)	Ever smoked cigarettes n (%)	Current cigarette smoker n (%)	P	Never used e-cigarettes n (%)	Ever used e-cigarettes n (%)	P	Never used HNB tobacco products n (%)	Ever used HNB tobacco products n (%)	P
**Drinking frequency**										
Non-drinker	45,953 (90.9)	3304 (6.7)	1116 (2.4)	< .001	48,520 (96.1)	1853 (3.9)	< .001	49,933 (99.0)	440 (1.0)	< .001
1–5 days	4610 (61.4)	1206 (16.1)	1621 (22.5)		5833 (77.4)	1604 (22.6)		6825 (91.4)	612 (8.6)	
6–9 days	453 (43.2)	160 (15.5)	426 (41.3)		649 (61.1)	390 (38.9)		841 (80.3)	198 (19.7)	
More than 10 days	484 (39.3)	148 (11.9)	559 (48.8)		672 (54.0)	519 (46.0)		873 (72.7)	318 (27.3)	
**Drinking quantity**										
Non-drinker	45,953 (90.9)	3304 (6.7)	1116 (2.4)	< .001	48,520 (96.1)	1853 (3.9)	< .001	49,933 (99.0)	440 (1.0)	< .001
≤2 bottles of beer	3821 (71.1)	788 (15.0)	716 (13.9)		4590 (85.3)	735 (14.7)		5107 (95.7)	218 (4.3)	
3–4 bottles of beer	1352 (44.0)	535 (16.5)	1196 (39.5)		1985 (63.5)	1098 (36.5)		2580 (82.9)	503 (17.1)	
>4 bottles of beer	374 (28.5)	191 (15.3)	694 (56.2)		579 (43.7)	680 (56.3)		852 (67.6)	407 (32.4)	
**Alcohol intoxication**										
No-intoxication	50,966 (86.6)	4615 (8.1)	2905 (5.3)	< .001	54,841 (93.3)	3645 (6.7)	< .001	57,317 (97.8)	1169 (2.2)	< .001
1–2 days	418 (35.4)	165 (13.3)	579 (51.3)		667 (55.5)	495 (44.5)		918 (78.1)	244 (21.9)	
3–4 days	56 (35.5)	19 (10.9)	93 (53.6)		78 (46.0)	90 (54.0)		118 (69.1)	50 (30.9)	
≥5 days	60 (27.9)	19 (9.1)	145 (63.0)		88 (39.1)	136 (60.9)		119 (53.9)	105 (46.1)	
**Alcohol availability**										
No-attempt	48,941 (89.3)	3956 (7.5)	1642 (3.3)	< .001	52,132 (95.2)	2407 (4.8)	< .001	53,941 (98.8)	598 (1.2)	< .001
Impossible	969 (68.5)	159 (12.3)	270 (19.2)		1142 (81.2)	256 (18.8)		1308 (93.4)	90 (6.6)	
Possible with effort	1008 (42.0)	426 (16.6)	975 (41.4)		1533 (62.4)	876 (37.6)		1994 (82.0)	415 (18.0)	
Possible without effort	582 (33.3)	277 (16.3)	835 (50.4)		867 (49.4)	827 (50.6)		1229 (71.7)	465 (28.3)	
**Drug use**										
Never	51,155 (85.4)	4724 (8.1)	3533 (6.4)	< .001	55,258 (92.4)	4154 (7.6)	< .001	58,002 (97.4)	1410 (2.6)	< .001
Ever	305 (59.6)	79 (17.0)	114 (23.5)		362 (70.9)	136 (29.1)		407 (80.1)	91 (19.9)	
Current	40 (32.2)	15 (10.4)	75 (57.5)		54 (40.0)	76 (60.0)		63 (45.4)	67 (54.6)	

^1^Weighted percentages following complex sample analysis.

HNB, heat-not-burn.

### Association of alcohol-related behaviors and drug use with ever having used e-cigarettes and HNB tobacco products

The associations of alcohol-related behaviors and drug use with eve having used e-cigarettes and HNB tobacco products among adolescents are shown in Table 3. In the multiple logistic regression model, which adjusted for gender, grade, perceived school performance, perceived economic status, and cigarette smoking status, ever having used e-cigarettes and HNB tobacco products was significantly associated with each alcohol-related behavior and drug use.

**Table 3:** Alcohol-related behaviors and drug use among Korean adolescents who had ever used e-cigarettes or HNB tobacco products (N = 60,040)^1^.

	Ever used e-cigarettes	Ever used HNB tobacco products
	AOR (95% CI)	AOR (95% CI)
**Drinking frequency**		
Non-drinker	1.00	1.00
1–5 days	1.96 (1.79–2.16)	1.92 (1.63–2.26)
6–9 days	2.61 (2.13–3.19)	2.95 (2.35–3.69)
More than 10 days	3.47 (2.80–4.31)	4.10 (3.20–5.27)
*P for trend*	< .001	< .001
*Nagelkerke R*^*2*^	.55	.47
**Drinking quantity**		
Non-drinker	1.00	1.00
≤2 bottles of beer	1.56 (1.38–1.76)	1.30 (1.06–1.61)
3–4 bottles of beer	2.64 (2.32–3.01)	2.75 (2.29–3.30)
>4 bottles of beer	3.85 (3.25–4.55)	4.16 (3.32–5.20)
*P for trend*	< .001	< .001
*Nagelkerke R*^*2*^	.55	.48
**Alcohol intoxication**		
No alcohol intoxication	1.00	1.00
1–2 days	2.11 (1.78–2.50)	1.85 (1.50–2.28)
3–4 days	3.97 (2.27–6.96)	3.11 (1.94–4.98)
≥5 days	4.03 (2.34–6.94)	5.74 (3.60–9.17)
*P for trend*	< .001	< .001
*Nagelkerke R*^*2*^	.54	.47
**Alcohol availability**		
Non-trial	1.00	1.00
Impossible	1.76 (1.45–2.15)	1.64 (1.23–2.18)
Possible with an effort	2.41 (2.11–2.77)	2.77 (2.30–3.34)
Possible without any effort	3.37 (2.88–3.95)	3.80 (3.17–4.55)
*P for trend*	< .001	< .001
*Nagelkerke R*^*2*^	.55	.48
**Drug use**		
Never	1.00	1.00
Ever	2.53 (1.75–3.65)	4.92 (3.31–7.29)
Current	4.21 (2.07–8.56)	12.17 (6.02–24.59)
*P for trend*	< .001	< .001
*Nagelkerke R*^*2*^	.54	.47

^1^Adjusted by gender, grade, self-perceived school performance, perceived economic status, and cigarette smoking status.

AOR, adjusted odds ratio; CI, confidence interval.

The higher ORs of ever having used e-cigarettes were 3.47 (95% CI: 2.80–4.31), 2.61 (95% CI: 2.13–3.19), and 1.96 (95% CI: 1.79–2.16), and the higher ORs of ever having used HNB tobacco products were 4.10 (95% CI: 3.20–5.27), 2.95 (95% CI: 2.35–3.69), and 1.92 (95% CI: 1.63–2.26) for participants who drank alcohol more than 10 days per month, six to nine days per month, and one to five days per month, respectively, than for non-drinkers, and the likelihood of ever having used e-cigarettes and HNB tobacco products increased with drinking frequency (P for trend < .001). The higher ORs of ever having used e-cigarettes were 3.85 (95% CI: 3.25–4.55), 2.64 (95% CI: 2.32–3.01), and 1.56 (95% CI: 1.38–1.76), and the higher ORs of ever having used HNB tobacco products were 4.16 (95% CI: 3.32–5.20), 2.75 (95% CI: 2.29–3.30), and 1.30 (95% CI: 1.06–1.61), for participants who drank more than four bottles of beer, more than two but no more than four bottles, and two or fewer bottles, respectively, than for non-drinkers, and the likelihood of ever having used e-cigarettes and HNB tobacco products increased with drinking quantity (P for trend < .001). In addition, the higher ORs of ever having used e-cigarettes were 4.03 (95% CI: 2.34–6.94), 3.97 (95% CI: 2.27–6.96), and 2.11 (95% CI: 1.78–2.50), and the higher ORs of ever having used HNB tobacco products were 5.74 (95% CI: CI: 3.60–9.17), 3.11 (95% CI: 1.94–4.98), and 1.85 (95% CI: 1.50–2.28), for participants who experienced alcohol intoxication more than five days per month, three to four days per month, and one to two days per month, respectively, than for non-drinkers, and the likelihood of ever having used e-cigarettes and HNB tobacco products increased with the frequency of alcohol intoxication (P for trend < .001). Specifically, taking the participants who did not try to buy alcohol products as a reference group, the higher AORs of those who bought alcohol products with no effort was 3.37 (95% CI: 2.88–3.95) for people who had ever used e-cigarettes, and 3.80 (95% CI: 3.17–4.55) for those who had ever used HNB tobacco products. Taking the participants who have never used drugs as the reference group, the higher AORs for participants who were current drug users and or had ever used drugs were 4.21 (95% CI: 2.07–8.56) and 2.53 (95% CI: 1.75–3.65) for those who had ever used e-cigarettes (P for trend < .001), and 12.17 (95% CI: 6.02–24.59), and 4.92 (95% CI: 3.31–7.29) for those who had ever used HNB tobacco products (P for trend < .001), respectively.

## Discussion

Our findings, based on a large representative survey of South Korean adolescents, revealed that regression models including alcohol and drug use behavior variables could significantly identify those who had ever used e-cigarettes and HNB products. The data from this sample suggest that the relationship of HNB tobacco products with alcohol and drug use is similar to that of e-cigarettes.

Previous studies revealed associations between e-cigarette use and risky behaviors such as alcohol and drug use among adolescents. E-cigarette users were more likely to engage in current drinking, current binge drinking, and marijuana use than never-smokers among US adolescents [37]. In a sample of North West England teenagers, drinking behaviors such as drinking to get drunk and alcohol-related violence were strongly associated with e-cigarette access, with this association being particularly strong among never-smokers [31]. Additionally, ever having used e-cigarettes was related to ever having used marijuana and ever-binge drinking among New Zealand adolescents [32]. E-cigarette users were more likely to use a variety of substances than were non-users in a sample of Icelandic students [38]. E-cigarette use was associated with binge drinking, marijuana use, illicit drug use, and nonmedical prescription drug use among US adolescents [39], and with cannabis use among Canadian high school students [40]. Congruent with previous studies, our results showed that ever having used e-cigarettes was associated with alcohol-related behaviors and drug use among Korean adolescents. In addition, there was a dose-response relationship for drinking frequency, drinking quantity, alcohol intoxication, alcohol availability, and drug use.

HNB tobacco products are expected to have an association with alcohol and drug use similar to that of tobacco and e-cigarettes. However, a longitudinal study conducted in Japan indicated that among HNB tobacco/e-cigarette never-users at baseline, current drinkers had lower odds ratios for predictors of current use of the HNB product I Quit Ordinary Smoking (IQOS) than did never-drinkers, and alcohol consumption and IQOS current use were not significantly associated [41]. Nevertheless, our study confirmed that ever having used HNB tobacco products showed the same positive association with alcohol and drug use as did ever having used e-cigarettes.

Adolescents with at-risk behaviors are prone to be attracted to novel products, and e-cigarettes appear to be a such a product; the reason adolescents who had never used e-cigarettes tried them initially was curiosity [32]. Although it is unclear what exactly evoked this curiosity, tailored adolescent substance use prevention programs that address multiple substances, including e-cigarettes and HNB tobacco products, are needed.

The interesting point of our study results was that a lower perceived economic status was associated with higher numbers of ever having used e-cigarettes and HNB tobacco products. Socioeconomic status has been widely known as an important determinant of cigarette smoking in adolescents, in that higher rates of conventional cigarette use are associated with lower SES [42–44]. Research has focused on the link between SES and e-cigarette use; however, the evidence is inconsistent [45–48]. It was found that lower socioeconomic backgrounds, such as low levels of parents’ education and higher unemployment, were associated with e-cigarette use among Finnish adolescents [45]. A recent study revealed that low SES was associated with past-month e-cigarette use among US adolescents [46]. However, some recent studies reveled that there was no significant association between SES and e-cigarette among adolescents [47, 48]. Despite the increased attention being paid to HNB tobacco products, no studies have been conducted to precisely examine the association between SES and HNB tobacco product use in adolescents. Among US and Japanese adults, there was no significant association between educational achievement and HNB tobacco product use [49, 50]. However, other recent studies showed that having an education at the tertiary level or above, a higher household income, and employment were associated with ever having used HNB tobacco products among Chinese adults in Hong Kong [51], and a higher level of education was associated with experimentation with HNB tobacco products [12]. Future research is needed to explore how SES impacts HNB tobacco product use in adolescents.

This study found that almost two thirds (65.2%) and one third (32.4%) of current smokers used e-cigarettes and HNB tobacco products, respectively. Considering that this study’s data collection was conducted just 11 months after HNB tobacco products were initially sold in the Korean market (May 2017), a rapid penetration of HNB tobacco products within a 1-year period can be inferred. Our findings also imply that e-cigarettes and HNB tobacco products were commonly used among those already using nicotine products and easily available to adolescents with an interest in smoking. Contrary to the insistence of tobacco companies that conventional cigarette smokers can convert into exclusive users of HNB tobacco products, almost all HNB tobacco products users were found to be dual or triple users of nicotine products, including conventional cigarettes and e-cigarettes [14]. The use of HNB tobacco products among adolescents would increase poly-use of other tobacco products [16]. Adolescents who use a broader range of tobacco products are more likely to have lower academic achievement and more likely to experience various risk factors, such as being cyberbullied, riding in a car with a drunken driver, or ever being arrested [52]. Among Hawaiian adolescents, dual users had higher risk of experiencing difficulties with academics and behavioral regulation [53]. Moreover, dual users among US teens were more likely to have ever drunk alcohol, currently drink alcohol, have ever tried marijuana, currently use marijuana, and have used drugs in their lifetime [54]. This suggests the need for continued tobacco regulatory efforts toward adolescents, including multiple forms of tobacco use, with strict regulation of new and emerging products.

Even though there was only a small proportion of never-smokers who had ever used e-cigarettes (1.4%) or HNB tobacco products (0.3%) in our study, e-cigarettes or HNB tobacco products used by never-smokers can act as a gateway product [55, 56]. Regular e-cigarette use among never smoking has increased among adolescents round the globe [57]. The number of never-smoking adolescents who used e-cigarettes had increased 3-fold in US from 2011 to 2013 [58]. It was reported that among ever e-cigarette users among US adolescents, 9.3% reported that they had never smoked conventional cigarettes [59]. A study of US adolescents reported that e-cigarette users who had never smoked cigarettes had greater interest in using tobacco products in the future than non-users of e-cigarette [58]. Some adolescents have their first nicotine experience with e-cigarettes, as the prevalence of e-cigarette use among adolescents who never smoked has increased [57–59], suggesting that the first nicotine experience with HNB tobacco products among adolescents might also increase. Similarly, an Italian sample demonstrated that HNB tobacco products may create nicotine-addicted generations, as the absolute number of those who had tried HNB tobacco products was almost the same as that of current smokers [12]. Using HNB tobacco products may result in adolescents not only initiating tobacco use but also increasing poly-use [16]. Thus, there is an urgent need to assist the prevention and control of e-cigarettes and HNB tobacco product use among never conventional cigarette smoker adolescents.

### Limitations

Our study has several limitations that must be considered. First, this was a cross-sectional study, so causal inferences cannot be determined. Further longitudinal studies are needed to understand the potential harm of alcohol and drug use as consequences of ever having used e-cigarettes or HNB tobacco products. Second, this study utilized a secondary data, so it was impossible for the current researchers to specify the variables of most interest. The use of e-cigarettes and HNB tobacco products was assessed with a single item, which asked participants to choose ever having used and never having used. The association of e-cigarette and HNB product use with alcohol or drug use might be different if use were measured as current use or frequency rather than dichotomously. Moreover, ever having used e-cigarettes was assessed via simple wording without any specific description, which may have led to exposure misclassification bias. In addition, this study could not clarify the e-cigarette products between nicotine-containing e-cigarettes and nicotine-free e-cigarettes. Further research needs to take into account the questions used to probe various variables and clearly present the question pertaining to each variable. Third, this study was based on self-report; therefore, self-presentation bias may exist. Adolescents may underreport their alcohol-related behavior or drug use because of social desirability; thus, drinking frequency, drinking quantity, alcohol intoxication, alcohol availability, or drug use might have been inaccurate.

### Strengths

To the best of our knowledge, this study is the first to utilize nationally representative data of South Korean adolescents to examine the association of ever having used e-cigarettes and HNB tobacco products use with alcohol-related behaviors and drug use. In addition, this study revealed a dose-response relationship between ever having used e-cigarettes and HNB tobacco products and drinking frequency, drinking quantity, alcohol intoxication, alcohol availability, and drug use.

### Implications and suggestions for future research

As a result of the aggressive marketing efforts of the tobacco industry, the popularity and use of various types of nicotine products among adolescents has increased [16, 60]. Our results suggest the possibility that adolescents who have ever used e-cigarettes or HNB tobacco products could be more vulnerable to alcohol-related behaviors and drug use. As multiple tobacco use rapidly increases among adolescents, health professionals need to assess and closely monitor the use of e-cigarettes and HNB tobacco products when implementing smoking cessation programs or other health promotion interventions related to substance use. Despite the rapid penetration and soaring popularity of HNB tobacco products, epidemiological or scientific evidence regarding these products is still limited. Governments, tobacco control authorities, and researchers should understand the marketing strategies and claims of the tobacco industry, and consider how to monitor and regulate these products effectively.
